# The Need for Realistic Behavior Modeling in Life Cycle Assessment

**DOI:** 10.1021/acs.est.5c15967

**Published:** 2026-07-27

**Authors:** Shelie A. Miller, Tamar Makov

**Affiliations:** † Center for Sustainable Systems, School for Environment and Sustainability, 1259 University of Michigan, Ann Arbor, Michigan 48109, United States; ‡ Guilford Glazer Faculty of Business and Management, 189155 Ben-Gurion University of the Negev, Be’er Sheva 8410501, Israel

**Keywords:** consumer behavior, use phase, spillovers, rebound effect, human–product interactions

## Abstract

Tackling complex environmental problems requires both technological and socio-cultural innovation. Life-cycle assessment methods rigorously and systematically analyze the environmental impacts of products, often focusing on technical parameters. Human behavior is rarely treated with the same level of nuance or complexity, despite having a major influence on the environmental impacts of many products. LCA must evolve to more accurately reflect the role of behavior in the environmental impacts of products. This Perspective provides a framework to identify important human–product interactions that can influence the results of an LCA. Heterogeneous consumer behavior regarding product substitution, use intensity, and lifespan is an important factor that should be explored beyond simple sensitivity analysis or scenario generation. We recommend increased data collection and interdisciplinary collaboration to assess actual consumer behavior, more explicit guidance for integrating consumer behavior into LCAs, and improved communication of behavior-dependent LCA results.

## Graphical abstract

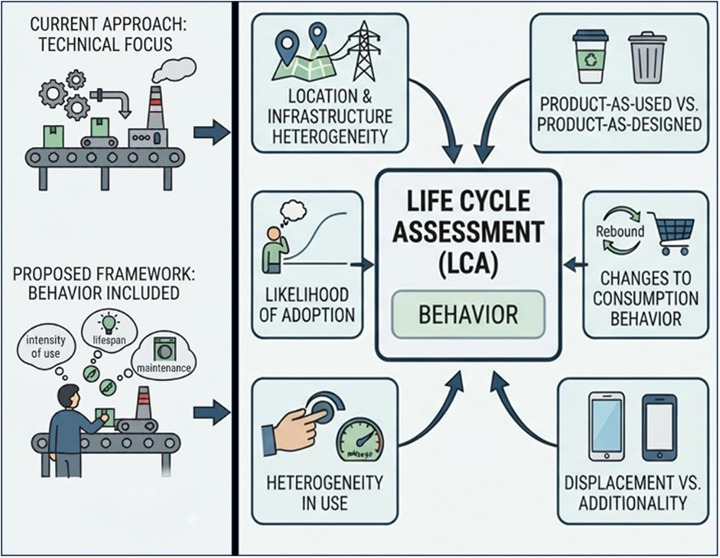

## Introduction

Human behavior can alter the life cycle impacts of many consumer products and services, from vehicles to clothing to packaging. Despite growing awareness of the myriad ways in which consumer behavior can affect LCA results,^1,2^ simplistic assumptions about product use intensity and lifespan, maintenance, replacement, and end-of-life (EoL) management remain common across industry and academic LCAs.^3–5^ A growing number of papers use consumer surveys, industry data, national statistics, and even behavioral experiments to inform LCA modeling.^6,7^ Nevertheless, LCA’s tendency to focus on the technical parameters of a system persists.^1,8^ Neglecting or greatly oversimplifying the role of the consumer may create systematic errors in LCA, either underestimating or overestimating environmental impacts.^1,2,5,7^

This Perspective advocates for greater and more rigorous integration of behavior in LCA and related approaches (e.g., material flow analysis). We propose a framework that identifies product sectors where behavior may be important and categorizes consumer attributes and behaviors that can alter the environmental impact of products. We are certainly not the first to advocate for more formal consideration of consumer behavior.^1,7,9–18^ To our knowledge, this Perspective is the first to try to systematize the different kinds of consumer behaviors relevant to LCA.

There are likely a variety of reasons that LCA has traditionally focused on technical parameters. First, the origins of LCA are rooted in engineering principles, using mass balance throughout the supply chain to systematically assess environmental impact. In addition, many LCAs are conducted from an industry perspective, often using a cradle-to-gate boundary that does not incorporate use or end-of-life phases where behavior is most important.^15^ Even when using a cradle-to-grave boundary, a firm’s ability to manage consumer behavior is limited, so focusing on controllable technical elements rather than attempting to influence behavior can make sense. When user behavior is included, it is often assumed to follow optimal conditions and design assumptions. For example, product category rules (PCR) under Environmental Product Declarations (EPD) may mandate technically oriented assumptions rather than actual use behavior to ensure a consistent approach (e.g., that apparel lifespan be modeled based on products’ washing durability based on performance testing).^19^

In addition, data from behavioral studies do not easily fit into the inventory data collection process. Methods to collect technical and behavioral data are often two distinct domains of inquiry, each employing different tools, methods, and approaches. While disciplines such as psychology, marketing, judgment, and decision-making study environmental behaviors, the literature is mostly focused on understanding why consumers engage (or do not engage) in sustainable behaviors and identifying effective methods to encourage those behaviors.^20–27^ Research on consumer behavior examines aspects such as motivations, norms, perceptions, willingness to pay, etc., with methods and results that are not easily translated into life cycle inventory parameters. Understanding why people behave as they do is essential to motivate behavior change; however, this level of detail is not necessary to better incorporate behavior into LCA.^20^ Human factors research is another discipline that has methods and tools useful to assess the intersection of behavior and environmental impact; however, most human factors research focuses on improved safety, ergonomics, or error reduction rather than environmental impact.^28^

Nevertheless, the influence of behavior on the LCA results can be as significant as any technical parameter.

Without readily accessible and compatible behavior data, both attributional and consequential LCAs often use average behavior or scenario analyses that present best- and worst-case assumptions to cover the full impact space. These average or extreme values may not capture nuanced or heterogeneous behavior patterns.^29^

Rather than oversimplifying behavior for the sake of cleaner modeling, it is essential to incorporate realistic, data-driven portrayals of behavior, even when doing so is inconvenient or resource-intensive. We provide numerous examples to demonstrate that consumer attributes (e.g., socioeconomic status, lifestyle) and behaviors (e.g., frequency of reuse, maintenance habits) can change a product’s environmental impacts. The degree of behavior’s importance naturally varies based on the product.

## Human–Product Interactions Framework

This framework provides structure and guidance to identify potentially relevant consumer attributes and behaviors to consider for a given LCA. It is not intended to be prescriptive or change any major elements of the LCA process. Behavior is relevant to many different research questions, so the framework is not specific to either attributional or consequential LCA, nor comparative or noncomparative LCA.

The extent to which behavior is relevant to an LCA is determined by the type of product, the purpose of the assessment, and the research questions considered. Behavior is relevant in a noncomparative attributional LCA of electric vehicles investigating how charging behavior influences battery life. It is also relevant in a comparative consequential LCA modeling market penetration of EVs across vehicle types. Meanwhile, behavior is not relevant for LCAs that focus on material selections independent of the car performance or driving behavior.

Although ISO 14040/14044 allows for this fit-to-purpose approach to LCA, it can run in conflict with standardization efforts, including Environmental Product Declarations and the EU Product Environmental Footprint methods. The conflict between flexibility and standardization in LCA is not unique in this context, although behavior modeling introduces greater uncertainty than that observed in many technical parameters.

Behavior is most obviously associated with the use and end-of-life phases but is relevant to many aspects of an LCA. For example, behavioral considerations can be important during boundary definition and selection of a functional unit. Narrow system boundaries can obscure the effects of consumer behavior. For example, insights from packaging LCA can be substantially different when the boundary includes food waste behaviors.^30^ Functional units can be selected that incorporate or exclude differences in behavior. For example, the functional unit for clothes washing may be defined as “per 5 cu. ft. load of laundry” or “per one month household clothes washing.” Depending on the goal and scope of an LCA, each of these functional units may be reasonable but account for behavior differently. The “per 5 cu. ft. loads of laundry” functional unit incorporates attributes that vary by household (e.g., appliance, settings, electricity grid differences) but neglects use behaviors captured by “per one month household clothes washing” (e.g., full capacity vs partial loads laundering frequency).

## Categories of Human–Product Interactions

The scope of an LCA differs according to its specific goals and contexts. As such, not every LCA requires an extensive consideration of human–product interactions. Although the use phase is most associated with behavior, even products without major use-phase impacts can be influenced by behavior.

The proposed framework identifies six major categories of human–product interactions that may be useful for LCA practitioners to consider, grouped by (i) consumer attributes and (ii) consumer behavior, subcategorized by direct and induced behaviors.

Table 1 defines, describes, and provides examples of each category, further explained below. This framework focuses on observed behaviors rather than delineating behavioral antecedents or underlying mechanisms that drive the behavior. Some of the categories are routinely and commonly included in LCA (e.g., infrastructure heterogeneity). Others are less commonly included yet may still alter LCA results. These categories provide organization and guidance for LCA researchers and practitioners looking to incorporate more realistic behavior modeling in their assessments, acknowledging that there can be some overlap between categories. The following section provides more detailed descriptions of the categories of human–product interactions.

**Table 1:** Summary of Major Human–Product Interactions and Examples

category	description	examples
Consumer Attributes		
location and infrastructure heterogeneity	differences in geography and environmental intensity of infrastructure (i.e., electricity, transport, water, waste management)	· influence of different electricity grids
		· last-mile for urban vs rural consumers
		· availability and types of electric vehicle charging infrastructure
		· availability of end-of-life infrastructure
		· different water infrastructure (e.g., municipal vs well water, desalination vs freshwater)
likelihood of adoption	prior behavior, attitudes, and opinions may determine whether a consumer is likely to adopt a product. Lower impact products may have higher likelihood of adoption by consumers that have a better-than-average environmental baseline.	· adoption of alternative proteins or food waste reduction efforts by vegetarians vs omnivores^31,32^
		· electric car purchases by owners of high efficiency hybrids vs average gas cars^33^
		· insulation installed in homes with high efficiency vs low efficiency HVAC
Consumer Behavior		
heterogeneity in use	consumers use products differently	· intensity and frequency of use (e.g., miles driven, frequency of laundering a T-shirt)^2^
		· product lifespan, maintenance, repair, and replacement rates
		· differences in end-of-life choices (e.g., landfill vs recycle, donate/reuse)
product-as-used vs product-as-designed	consumer behavior may differ from environmentally optimal (or expected) use	· human override of smart buildings features, leading to underperforming expectations^34^
		· treating reusables (e.g., shopping bags, water bottles, coffee cups) as semidisposable, only using a few times rather than design lifetime^35^
		· using disposable products multiple times^12^
		· suboptimal replacement rate of appliances/vehicles
		· consumer discards product unexpectedly, either not fully using a product (e.g., personal care item, replacing functional appliances due to renovations) or not engaging in expected repairs^5^
changes to consumption behavior	spillover behaviors outside of the primary analysis. This may include rebound effects, moral licensing, and product adoption that enables other consumption	· participation in e-commerce changes shopping behavior (e.g., amount of consumption, basket composition, return behaviors)^36^
		· influence of household refrigeration availability on increased meat consumption^37^
		· rebound effects such as increased fuel economy leading to increased vehicle miles
		· availability of recycling leads to greater overall consumption of materials^38^
displacement vs additionality	consumers may opt to increase overall consumption rather than replace older products	· E-scooter adoption displacing walking trips vs vehicle trips^39^
		· second-hand markets do not necessarily reduce purchases of new items^40^
		· Reused/recycled clothes or fabrics does not lead to reduced consumption and production^41^
		· keeping older refrigerators in garages or basements after purchasing a new model.
		· smartphones displacing multiple products of different functions (cell phones, digital cameras, planners)^42^

## Consumer Attributes

Consumer attributes are characteristics of a consumer that influence the environmental impact of a product. We define two main categories of consumer attributes: (i) location and infrastructure heterogeneity and (ii) likelihood of adoption.

### Location and Infrastructure Heterogeneity

Differences in the geography and background infrastructure can lead to different LCA results. Two central examples of infrastructure heterogeneity are differences in regional electricity grids and transportation distances. Regional water scarcity is also an important factor to consider for water-intensive products. Product performance can also vary in different climates (e.g., heat pumps). Additional factors to consider include local regulations surrounding building codes, the availability of community recycling programs, and the charging infrastructure for electric vehicles.

This factor is already well-recognized in LCA as an important consideration in LCA literature; it is often incorporated when relevant, particularly in spatially explicit LCA.^43^ Regionalized data for electricity and water production are commonly available.

### Likelihood of Adoption

Consumer adoption of new products varies according to affordability, peer influence, environmental attitudes, and a range of other factors.^44,45^ While convenient, using a market average can neglect differences among consumer segments and skew LCA results when adoption is not uniform across the population.^11,46^ If customers with pro-environmental attitudes are more likely to purchase lower-impact products, LCA studies will overestimate the improvement potential of green product alternatives when based on population averages.^47^

As an example, an owner of an inefficient car with an internal combustion engine (ICE) will realize greater environmental benefits adopting an electric car than an owner of a highly efficient hybrid. Although some studies do take this into account,^33^ LCAs that compare electric cars to the overall ICE vehicle fleet may overestimate emission savings. As another example, an omnivore who substitutes a beef burger with a plant-based burger realizes greater environmental benefits than a strict vegetarian who eats the same burger.^32^

Baseline characteristics are important to consider whenever there is significant heterogeneity across consumer baselines, particularly for comparative attributional or consequential LCAs.

## Consumer Behaviors

Consumer product behavior includes direct-use behaviors as well as changes in consumption patterns. The four categories include (i) heterogeneity in use, (ii) product-as-used vs product-as-designed, (iii) changes to consumption behavior, and (iv) displacement vs additionality.

### Heterogeneity in Use

Consumers’ interactions with products vary. Simplified behavior assumptions can obscure the influence consumer choices can have on environmental impact. Specific considerations include different product waste rates for consumable products (e.g., food, cosmetics), end-of-life choices, operating and maintenance patterns, and product lifetime. Without an understanding of how consumers use a product in practice, the results of LCA are likely to be inaccurate.

As an example, product repair is often modeled using a distribution of repair frequency or taking a weighted average of consumers who repair products. In reality, the environmental impacts of this product are bimodal, depending on whether it is repaired or discarded. Simple reporting of weighted averages does not appropriately communicate the agency that a consumer has to determine the environmental impact of a product.

Most consumer-oriented products may benefit from improved attention to heterogeneity. Examples range from choices around thermostat settings, intensity of wearing and washing clothes, maintenance, reuse, repair behavior, and product lifetime. Unfortunately, quality data regarding actual consumer use behavior are scarce for many product categories.

### Product-as-Used vs Product-as-Designed

LCA practitioners often make the simplifying assumption that consumers will use products as they are intended rather than accounting for actual consumer behavior. In practice, there are many examples where consumers do not use products optimally.

To illustrate, a study of reusable bottles found them to be more environmentally benign compared to single-use PET bottles, using an assumption that the stainless-steel bottle will be refilled 1,200 times.^3^ While it is technically possible to hold on to the same bottle and refill it daily for 2-3 years, in retrospect, we question whether this was a realistic assumption.^11^ Product lifespan is often modeled based on functional durability or a market average, potentially masking significant variability within the same product category.^48^

One important subcategory of direct use is waste-related behaviors, specifically if the consumer discards a portion of the product before it is used for its intended purpose. Food waste is a particularly important example, although essentially any consumable product that goes to waste rather than fulfilling its intended purpose fits into this category.

### Changes to Consumer Behavior

Some products have the ability to change how consumers behave, which may not always be reflected in an LCA. In some cases, changing behavior can be taken into account by adjusting the functional unit. For example, consumers may drink more of a beverage when it is packaged in a 20 oz bottle rather than a 16 oz container. Changing the functional unit from “16 oz. of beverage consumed” to “beverage consumed in one sitting” addresses this behavior change. Similarly, online shopping causes changes to purchasing behavior that are still not fully understood. The results of an analysis differ when calculated using functional units of per product, per basket size, or per month of consumption.^49,50^ Selecting appropriate functional units should be sufficient to account for this factor, although in practice, induced consumption is not often estimated and incorporated into the analysis.

One well-documented example of an induced behavior change is the rebound effect, the phenomenon where increased consumption follows improved efficiency.^51^ For example, lower costs of operating an EV may increase the overall distance driven. In addition, zero-waste events and households with recycling tend to consume more overall, perhaps because they are provided signals that their consumption activity has been mitigated.^38,52^ Although the rebound effect is well documented and discussed in the sustainability literature, it is rarely incorporated within LCA.^51^ When rebound effects are included in LCA, the approach is often inconsistent.^53^

### Displacement vs Additionality

Many comparative LCAs presume a displacement effect, where an older product is retired when a new one is adopted. In reality, customers may retain the older product while also acquiring a new product.^5,40,54^ For example, a garage fridge is the colloquial term for an older refrigerator kept in use to store overflow and discretionary food and beverages. The purchase of an energy-efficient refrigerator will not result in net household benefits if the older, less efficient refrigerator remains in use, even though that may be implicitly assumed within a comparative LCA.^5^ Multifunctional products have similar potential issues when LCA assumes displacement of multiple individual products that does not occur.^55^ Similarly, an LCA that assumes a second-hand purchase avoids production of a new product may not be realistic.^5^ Consuming second-hand clothing is correlated with greater purchases of new clothing.^56^

LCA may also incorrectly assume which products are displaced. For example, alternative proteins may be compared to beef but may ultimately reduce other sources of protein such as other plant-based products or fish and seafood.^32^ Whenever the environmental benefits of a product hang on it replacing an existing one, it is not enough to explore adoption rates but also disadoption of incumbent products.^47^

## Applying the Framework

To provide concrete examples of how the framework may be useful, Table 2 provides example research questions for two products, each with different types of potentially relevant behaviors. Appropriate research questions depend on the specific goal and scope of the study; these are merely illustrative examples.

**Table 2:** Example Questions for a Plant-Based Meat Alternative and a Heat Pump Clothes Dryer

	example research questions	
category	plant-based meat alternative	heat pump clothes dryer
Attributes		
infrastructure heterogeneity	Is the product broadly available and accessible?	How do different regional electricity mixes impact results?
baseline consumer environmental attributes (who will adopt the product)	What is the fraction of vegetarians and omnivores who purchase the product?	Does the appliance appeal primarily to customers who already own an energy efficient machine?
Behaviors		
heterogeneity in use	Is food waste composted or landfilled?	Are there changes to laundering frequency or timing (e.g., peak vs off-peak electricity) due to longer drying cycles?
product use vs product as designed	Are there greater food waste rates due to lack of experience with the product?	Is the appliance properly maintained throughout its life and replaced according to expected lifetime assumptions?
changes to consumption behavior	Does eating plant-based change overall food consumption habits?	Are there observable rebound effects?
displacement vs additionality	Does it displace red meat at 1:1? Did the household previously consume primarily vegetarian? What other types of foods does it displace?	Did the household previously line-dry clothes or did they replace an existing dryer?

## Recommendations for Future Research

This Perspective argues that there is a strong need for the LCA community to better incorporate human behavior explicitly into LCA. We provide three recommendations for future research and practice to improve the robustness of LCA in its treatment of human–product interactions: (1) goal and scoping phases of LCA should explicitly identify behaviors that may influence results and methodological choices surrounding behavior; (2) greater efforts should go into collecting behavior data and making prior research available in formats accessible to the LCA community, likely requiring collaborations with behavioral scientists; (3) the importance of behavior should be explicitly identified during the interpretation phase and better communicated when reporting results.

First, guidelines for researchers to explicitly address behavior considerations should be developed using robust methods to translate research on consumer behavior into the LCA framework. Even when an analyst recognizes that human behavior is important, there is no clear guidance on how to incorporate more complex behavioral concepts.

Second, more data are needed regarding user behavior. Even when an LCA practitioner wishes to better incorporate human behavior, quality consumer data are expensive and scarce. There is a need to develop better, easily accessible databases containing behavioral data. Although we recognize that behavioral research is typically not conducted with the goal of life-cycle database integration, it is still possible to glean relevant information via existing surveys, behavioral experiments, or digital metadata. Tools such as conjoint analysis can be used to estimate adoption as well as displacement rates, along with market data at both the macro- and household levels, which can also be used to define how costs affect behaviors. Segmentation and clustering analyses can help establish relevant baselines and incorporate the heterogeneity present across the population. Particularly for digital products and services relaying on ICT (e.g., the sharing economy), OMEs and service providers have in many cases data on actual use intensity, use duration, maintenance practices, repair, and more. This is true for consumer electronics but also increasingly for white goods, appliances, office equipment (e.g., printers), cars, and other products now connected to the Internet of Things, as well as services relying on ICT such as the sharing economy, tourism and leisure, and media consumption.

There is also a need for more data collection for environmentally important behaviors, which will likely require greater collaboration between LCA practitioners and the social science communities. Identifying specific behavioral research methods useful to generate data relevant to LCA is outside the scope of this Perspective, although some are described in a recent review paper.^13^ We believe that this is an area ripe for future exploration that can build on prior studies.

Finally, there is a need to change the way LCA results are presented that do not oversimplify the role of the consumer in determining the environmental impact of a product. Improved communication acknowledges that consumer behavior can lead to different LCA outcomes, particularly when the directionality of results can be reversed due to behavior. Rather than blanket statements such as “Alternative A is superior to Alternative B,” more correct messaging may be “Alternative A has lower impact *if and only if* it is used in manner X”. In addition, to improved scientific validity, this kind of messaging highlights the agency of the consumer in affecting environmental impact.

There is much work remaining to better integrate behavior into the LCA. This Perspective seeks to highlight the importance of explicitly including behavior in LCA, provide specific examples of where it has been done well, and offer some guiding questions and tools to help identify relevant research questions.
